# Effortless Fabrication of Nanofused HKUST-1 for Enhanced Catalytic Efficiency in the Cyanosilylation of Aldehyd

**DOI:** 10.3390/ma18051131

**Published:** 2025-03-02

**Authors:** Tian Zhao

**Affiliations:** School of Chemical Engineering and Technology, Sun Yat-sen University, Zhuhai 519082, China; zhaot33@mail.sysu.edu.cn

**Keywords:** metal-organic frameworks, HKUST-1, nanofused structure, cyanosilylation of aldehydes, enhanced catalytic efficiency

## Abstract

HKUST-1 (HKUST = Hong Kong University of Science and Technology) is one of the most recognized metal-organic frameworks (MOFs) based on copper and trimesate, extensively studied for a variety of applications, such as gas storage, separation, adsorption, electrocatalysis, drug delivery, sensor and photodegradation, etc. In this work, we introduce a novel nanofused HKUST-1, referred to as N-CuBTC (BTC = trimesate), which has been synthesized with the hydrothermal method at room temperature (typical synthesis temperature is from 80~120 °C). The resulting N-CuBTC features an irregular particle morphology, with numerous crystals clustering together and edges that have fused, creating a hierarchical pore structure. In contrast to the traditional micro-sized octahedral HKUST-1 (named as M-CuBTC), N-CuBTC displays a unique clumped morphology, where the HKUST-1 crystals are seamlessly integrated into a cohesive structure. This innovative formation significantly enhances mass transfer capabilities and porosity accessibility. Consequently, N-CuBTC demonstrates markedly improved catalytic performance in the cyanosilylation of aldehydes.

## 1. Introduction

Metal-organic frameworks (MOFs) represent a class of porous materials, uniquely formed from the combination of inorganic metal ions with organic ligands [1,2]. These materials are characterized by their remarkably high specific surface areas, customizable pore structures, and the presence of unsaturated metal sites [3,4]. Such features bestow them with outstanding capabilities across a variety of applications, including catalysis, drug delivery, adsorption, and energy storage and conversion [5,6].

HKUST-1, in particular, is a notable three-dimensional porous structure composed of copper ions and trimesate ligands, represented by the empirical formula 3D-[Cu_3_(btc)_2_(H_2_O)_3_], where btc refers to trimesate [7,8]. Due to its straightforward synthesis process, high porosity, and impressive specific surface area (exceeding 1000 m^2^ g^−1^), along with its stability and resistance to water, HKUST-1 has emerged as one of the most extensively researched MOFs [9,10]. Its exceptional properties make it an ideal candidate for a range of applications, including gas storage, gas separation, catalysis, drug delivery, and various sensing technologies [11,12]. Recent studies have centered on the development and utilization of hierarchically porous HKUST-1, also known as HP-HKUST-1 [13,14,15]. The techniques employed to fabricate these hierarchical structures, which primarily include etching [16,17], modulation [18,19], and mixed-linker strategies [20], frequently lead to the creation of mesopores and macropores. However, these larger pores can be interpreted as defects within the otherwise uniform micron-sized HKUST-1 crystals. As a result, the HP-HKUST-1 obtained through these methods often exhibits reduced structural integrity, primarily manifesting as a decrease in crystallinity and a lower specific surface area [21,22]. Thus, there is an evident need for a simpler, more efficient, and economically viable template-free approach to synthesize fully connected HP-HKUST-1.

This research introduces an innovative technique for the fabrication of a fully nanofused structural variant of HKUST-1, which is referred to as N-CuBTC. This method exploits the alteration of the copper source used in the synthesis. Remarkably, under optimal conditions, using copper acetate as Cu source at ambient temperature and stirring for 24 h, the crystals of HKUST-1 can be reduced to the nanoscale while achieving complete fusion, leading to a nanofused structure characterized by entirely interconnected pore channels, known as N-CuBTC. When compared to the original micron-sized HKUST-1 (referred to as M-HKUST-1), the resulting hierarchical pore structure in N-CuBTC exhibited remarkable accessibility. This enhanced accessibility plays a significant role in improving its catalytic efficiency, particularly in the cyanosilylation reactions of aldehydes.

## 2. Materials and Methods

### 2.1. Raw Materials and Reagents

Cu(NO_3_)_2_·3H_2_O (99.5%, AR), Cu(CH_3_COO)_2_·H_2_O (99.5%, AR), trimesic acid (C_9_H_6_O_6_, 99.5%, AR), ethanol (C_2_H_5_OH, 99.5%, AR), benzaldehyde (C_7_H_6_O, 99+%, AR), trimethylsilylcyanide (Me_3_SiCN, 97%, AR), heptane (C_7_H_16_, 99%, AR), and hexadecane (C_16_H_34_, 99%, AR) were utilized in this study. Water with a residual conductivity of 0.1 μS/cm was obtained using a GRO-10L water purification system. All chemicals were sourced from Shanghai Aladdin Biochemical Technology Co., Ltd. (Shanghai, China) and were used as received without additional purification.

### 2.2. Sample Synthesis and Purification

Synthesis of conventional octahedral HKUST-1 (M-CuBTC): In this procedure, 1.21 g of copper nitrate trihydrate (equivalent to 5 mmol) and 0.63 g of trimesic acid (which corresponds to 3 mmol) were each individually dissolved in 15 mL of distilled water. Following this, the two solutions were combined and stirred (1000 r/min) continuously for a duration of 24 h at room temperature (25 °C). After thorough mixing, the resulting blue crystals were isolated using centrifugation. These crystals were then washed with 30 mL of anhydrous ethanol through ultrasonic agitation for 1 h, with the ethanol being replaced every 30 min. Once the washing procedure was completed, the product was again collected via centrifugation and subsequently placed in a vacuum drying oven set to 80 °C to dry overnight.

Synthesis of nanofused HKUST-1 (N-CuBTC): For the preparation of N-CuBTC, 1.00 g of copper acetate monohydrate (5 mmol) was dissolved in 15 mL of deionized water, while 0.63 g of trimesic acid (3 mmol) was dissolved separately in another 15 mL of deionized water. The two prepared solutions were then mixed and stirred (1000 r/min) for a continuous 24 h period at room temperature (25 °C). After stirring was complete, the blue product was collected by centrifugation. The purification of N-CuBTC was carried out following the same protocol utilized for M-CuBTC.

Cyanosilylation of benzaldehyde

A total of 425 mg (4 mmol) of benzaldehyde, along with 794 mg (8 mmol) of trimethylsilylcyanide (TMSCN), and 10 mg of the MILs catalyst were mixed in 15 mL of heptane, with 1 mL of hexadecane serving as the internal standard [23]. The reaction mixture was stirred at a temperature of 313 K, and its progress was periodically assessed using an Agilent Technologies 7890A gas chromatography (GC) system.

### 2.3. Detection and Characterization

#### 2.3.1. X-Ray Diffraction (XRD)

The samples were analyzed using a Bruker D8 Advance X-ray diffractometer (Bruker, Karlsruhe, Germany) equipped with a Cu target and Kα radiation (*λ* = 1.54182 nm), operating at a voltage of 30 kV. The measurement was conducted over a 2 h duration within a 5° to 80° test angle range. The XRD was used for phase identification of the samples.

#### 2.3.2. Surface Area and Pore Volume Measurement

The specific surface area and pore volume of the samples were determined using a specific surface area analyzer (NOVA-4200e, Quantachrome, Boynton Beach, FL, USA). Before the analysis, all samples underwent a standardized pretreatment, which included drying in a vacuum at 120 °C for 2 h. The chosen temperature of 120 °C and the duration of 2 h for the vacuum drying pretreatment were specifically selected to effectively remove the crystallization water from the samples. This temperature is sufficiently high to promote the evaporation of water without compromising the structural integrity of the material. The 2 h duration ensures thorough dehydration while preventing potential thermal decomposition or alteration of the sample’s properties. The measurements were carried out within a pressure range of 10.1325 to 101.325 kPa, employing high-purity nitrogen (N_2_) as the adsorbent at a temperature of 120 °C.

#### 2.3.3. Transmission Electron Microscopy (TEM)

The particle size and microscopic morphology of the samples were investigated using transmission electron microscopy (TEM, Talos F200X, FEI, Hillsboro, OR, USA) with an FEI Talos F200X instrument (Thermo Fisher Scientific, Waltham, MA, USA). For the examination, samples were dispersed in ethanol and then deposited onto a standard copper grid.

#### 2.3.4. Scanning Electron Microscopy (SEM)

To assess the microscopic morphology and particle dimensions, we employed a Zeiss Gemini 300 scanning electron microscope (SEM, Gemini 300, Zeiss, Jena, Germany). Prior to the imaging process, samples were prepared by placing them on a conductive gel substrate and coating them with a layer of gold to enhance conductivity.

#### 2.3.5. Fourier Transform Infrared Spectroscopy (FTIR)

Infrared spectra of the samples were obtained using an infrared spectrometer (Vertex 80 V, Bruker, Karlsruhe, Germany) over a wavenumber range spanning from 400 to 4000 cm^−1^. The resolution during the FTIR scanning was set at 1 cm^−1^.

#### 2.3.6. Gas Chromatography (GC)

The products resulting from the catalytic reaction were identified and analyzed utilizing an Agilent Technologies 7890A gas chromatography (GC, Agilent, Palo Alto, CA, USA) system.

## 3. Results and Discussion

**Figure 1:**
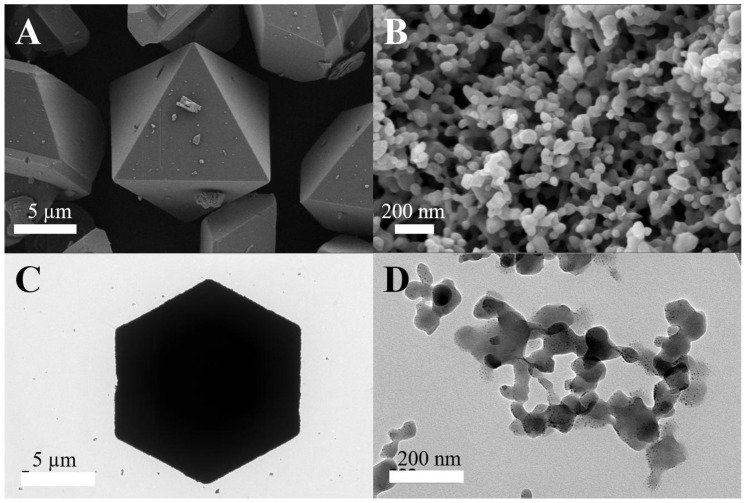
SEM images of (**A**) M-CuBTC and (**B**) N-CuBTC. TEM images of (**C**) M-CuBTC and (**D**) N-CuBTC.

The morphologies and structural characteristics of M-CuBTC and N-CuBTC were investigated through electron microscopic imaging, as illustrated in Figure 1. The M-CuBTC, serving as the control sample, displayed the quintessential octahedral crystalline form on a micron scale (refer to Figure 1A,C), featuring a consistent crystal size of approximately 12 μm across the sample.

In contrast, the N-CuBTC exhibited dramatically different features, showcasing aggregates of irregularly shaped nanocrystals, each measuring approximately 50 nm. These nanocrystals displayed significant evidence of intergrowth and fusion (see Figure 1B,D). It was observed that approximately ~100% of the nanocrystals were fully fused together (Figure 1B,D). Additionally, surface area measurements revealed that the specific surface area of N-CuBTC was only decreased by approximately 70 m^2^/g compared to the non-fused counterparts (M-CuBTC, Table 1). This unique arrangement enhances the material’s potential for various applications due to its improved accessibility and porosity.

It is clear that the samples obtained from different copper precursors exhibit notable differences in their morphology and structural characteristics. It has been established in prior studies that the type of copper source employed plays a crucial role in determining the nucleation dynamics and size distribution of the resulting metal-organic framework (MOF), HKUST-1. Specifically, the utilization of copper nitrate has been associated with the formation of larger HKUST-1 particles due to its slower nucleation rate. In contrast, the employment of copper acetate leads to the generation of nano-sized HKUST-1 particles, as the accelerated nucleation process facilitates a rapid formation of numerous smaller particles [24,25].

In the current investigation, it is proposed that the nanofusion mechanism contributing to the formation of N-CuBTC nanocrystals adheres to this established pattern. When copper acetate is used as the precursor, a swift generation of nanocrystallites occurs, which significantly increases the likelihood of nanofusion owing to the heightened surface energy of these small particles. The elevated surface energy results in stronger particle interactions, thereby promoting their fusion into larger aggregates [24,25]. Moreover, it is reported that the application of rapid stirring during the synthesis process can further facilitate the fusion of MOF nanoparticles [26]. This dynamic mixing likely enhances the opportunities for particle collisions and fusion, thereby contributing to the final characteristics of the synthesized N-CuBTC nanocrystals. Conversely, in reactions involving copper nitrate, the gradual nucleation rate leads to a different particle morphology observed in the M-CuBTC samples, where larger aggregates are formed without the same level of rapid surface interactions. In conclusion, the combined effects of precursor selection, nucleation dynamics, and external factors such as stirring are crucial in dictating the morphology and size of the synthesized nanocrystals, confirming that similar mechanisms are operational in the formation of N-CuBTC.

**Figure 2:**
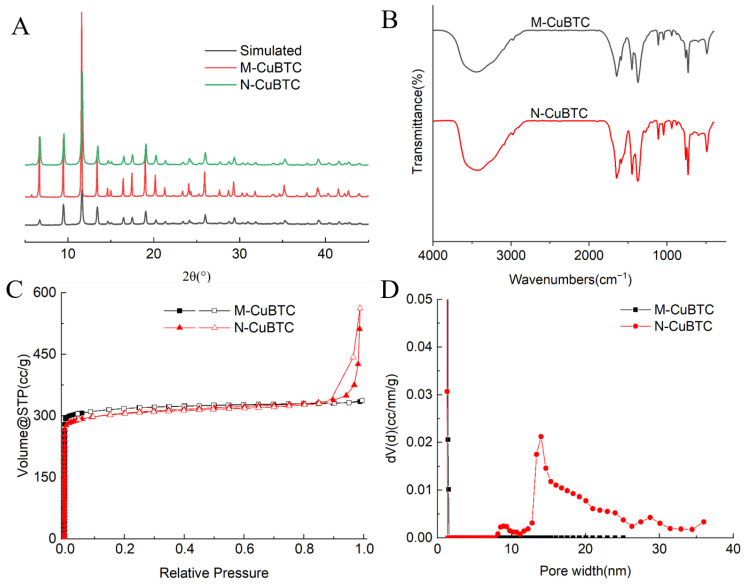
(**A**) XRD patterns and (**B**) FTIR spectra of M-CuBTC and N-CuBTC. (**C**) N_2_ adsorption–desorption curves and (**D**) NLDFT pore size distribution (PSD) curves for M-CuBTC and N-CuBTC.

The X-ray diffraction (XRD) analysis of the samples provided further insights, revealing that both M-CuBTC and N-CuBTC closely corresponded to the simulated XRD pattern of HKUST-1. This confirmed the purity of the phase for all samples studied (refer to Figure 2A). Notably, as we transitioned from M-CuBTC to N-CuBTC, a discernible decrease in the peak intensities of the XRD patterns was observed, along with a gradual softening of peak shapes (see Figure 2A). This trend suggests a reduction in particle size, which aligns well with the observations made through electron microscopic imaging, reinforcing the contrast between the two materials and their respective structural characteristics.

The application of Fourier transform infrared spectroscopy (FTIR) allowed us to investigate the coordination of trimesate with Cu^2+^ ions in the HKUST-1 framework, yielding results that align well with the early literature [27] (Figure 2B). The absorption peak observed at 730 cm^−1^ does indeed correspond to the presence of Cu ion clusters within the crystal structure, and it aligns well with the reference spectra reported in the literature. This consistency supports the accuracy of the peak assignments and further validates the findings regarding the structural characteristics of the material [28]. Furthermore, the spectra exhibited absorption peaks in the range of 1300 to 1700 cm^−1^, indicative of C-O vibrational modes associated with the carboxylic functional groups. Specifically, the peak at 1451 cm^−1^ was linked to the symmetric stretching vibration of the O=C-O bond, while the peak at 1634 cm^−1^ corresponded to the asymmetric stretching of the same bond.

Additionally, the peaks at 1374 cm^−1^ and 1573 cm^−1^ represented vibrational modes of C=C, signifying the successful intercalation of the BTC ligand within the HKUST-1 structure. A broad absorption peak situated at 3421 cm^−1^ indicated H-O stretching vibrations, which suggests a considerable presence of adsorbed water molecules within the HKUST-1 matrix. These spectral data imply that the coordination of the H_3_btc ligand with Cu^2+^ occurs in a bidentate fashion, resulting in the formation of the [Cu_2_(COO)_4_] structural unit, commonly referred to as the pulley unit. This structural motif has the potential to be activated through heating or under vacuum, thereby generating a substantial number of unsaturated coordination sites of Cu^2+^, which could function as catalytic Lewis acid sites.

The nitrogen adsorption–desorption isotherms for both M-CuBTC and N-CuBTC are presented in Figure 2C, with the accompanying porosity parameters provided in Table 1. The nitrogen adsorption–desorption isotherms for both M-CuBTC and N-CuBTC are indeed classified as typical Type I (a) isotherms. This classification indicates the presence of microporosity within the materials, highlighting their ability to adsorb nitrogen at low pressures [29]. The adsorption and desorption curves for M-CuBTC closely overlapping, demonstrating minimal hysteresis consistent with the characteristics of microporous materials. In contrast, the isotherms for N-CuBTC displayed noticeable hysteresis loops between the adsorption and desorption phases in the P/P_0_ range of 0.9 to 1.0, a behavior typical of meso- and macropores exist in the material [29] (refer to Figure 2C).

The pore size distribution (PSD) analyses for both M-CuBTC and N-CuBTC, calculated using nonlocal density functional theory (NLDFT) with a “slit-pore model,” are illustrated in Figure 2D. It is noteworthy that N-CuBTC contains not only its intrinsic micropores but also additional mesopores and macropores, confirming the observations made through electron microscopy. Conversely, M-CuBTC comprises exclusively micropores. This distinct difference suggests that N-CuBTC may offer considerable advantages in applications requiring swift mass transport, such as various catalytic processes.

The presence of coordinated water molecules within the HKUST-1 structure can be effectively removed when subjected to vacuum conditions, resulting in the formation of active Lewis acid sites [30,31]. This characteristic renders HKUST-1 a valuable catalyst for a variety of chemical reactions, including the cyanosilylation process involving aldehydes [32].

**Figure 3:**
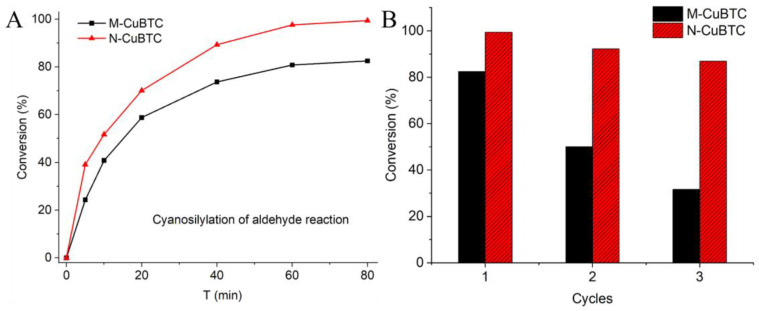
(**A**) Time-dependent conversion of benzaldehyde using M-CuBTC and N-CuBTC. (**B**) Comparison of conversions achieved with M-CuBTC and N-CuBTC over three reaction cycles.

**Scheme 1:**
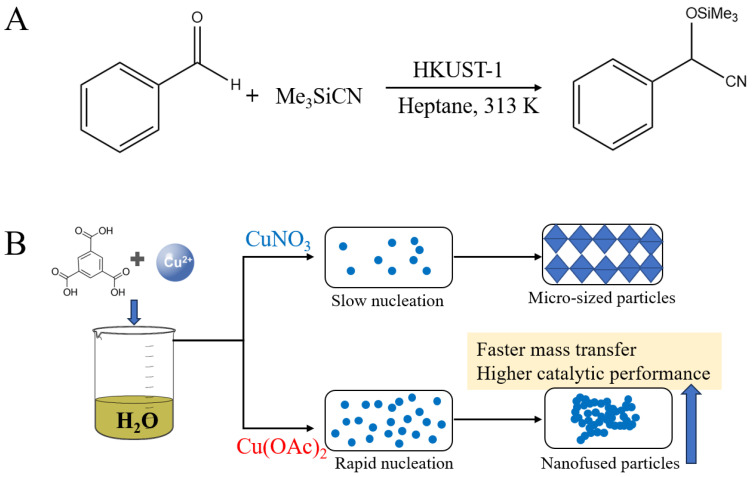
(**A**) The schemes of cyanosilylation of aldehyde reaction. (**B**) The synthesis procedure and possible mechanism for M-CuBTC and N-CuBTC.

To evaluate the catalytic performance of the synthesized materials M-CuBTC and N-CuBTC, reactions were conducted based on this methodology, with the reaction scheme illustrated in Scheme 1A. The results regarding the catalytic efficiency of both M-CuBTC and N-CuBTC are compiled in Table 2, while the substrate conversion over time is depicted in Figure 3A. Specifically, during the cyanosilylation of benzaldehyde, N-CuBTC achieved a remarkable conversion rate of 99.4%, which indicates a substantial 20% improvement over the performance of M-CuBTC as shown in Figure 3A and summarized in Table 2. Thus, N-CuBTC displayed considerably enhanced catalytic activity compared to M-CuBTC. Also, compare to other work, N-CuBTC exhibited quite good efficient and reusability (details please see Table 2).

Furthermore, N-CuBTC displayed impressive reusability in catalytic applications; after undergoing three reaction cycles in the cyanosilylation of benzaldehyde, it maintained a conversion rate exceeding 85% (see Figure 3B). In contrast, the performance of M-CuBTC as a catalyst deteriorated significantly after three cycles, with conversion dropping to below 40% (as illustrated in Figure 3B).

These findings clearly indicate that the distinctive nanofused hierarchical structure of N-CuBTC plays a critical role in enhancing its catalytic performance. The fully interconnected hierarchical pores increase the accessibility of the active sites located within the structure, facilitating improved mass transfer rates for the reacting substrate [33,34], which in turn significantly boosts the overall catalytic efficiency (refer to Scheme 1B) [35].

**Table 2:** Summary on catalytic performances for M-CuBTC/N-CuBTC and the comparison with other work.

Run	Catalysit	Reaction	Advantages & Disadvantages	Conversion (%)	Reference
1	M-CuBTC	Cyanosilylation of aldehyde reaction	highly efficient and reusability	82.5	This work
2	N-CuBTC			99.4	
3	[Zn(H_2_L_2_)]_2_		efficient, versatile/ long time	97	[36]
4	3-AC		efficient and reusability/surface modification limitations	94.3	[37]
5	[Co(H_2_L_1_)]_2_		efficient, non-solvent/moderate yield	92	[38]
6	MnI/DAPTA		efficient, green solvent/metal dependency	99	[39]
7	Co complexe		versatile/low efficient	77.4	[40]
8	F-MIL		efficient/limited-reusability	98.4	[41]
9	L2 + Ph_3_PO		high enantioselectivity/challenging optimization	71	[42]
10	TBD		efficient, low-catalyst-loading/	98	[43]
11	PF6		efficient/limited-to-aldehydes	99	[44]
12	Cu(ClO_4_)_2_·6H_2_O		efficient/electronic-effect-sensitive	98	[45]

## 4. Conclusions

In conclusion, this research successfully illustrates the synthesis of an innovative nanofused variant of HKUST-1, designated as N-CuBTC. This achievement was made possible by altering the copper precursor during the hydrothermal synthesis process. The resulting N-CuBTC displayed a sophisticated hierarchical porous architecture, which markedly improved its catalytic efficacy in the cyanosilylation of aldehydes. The catalyst attained an impressive conversion rate of 99.4% along with a turnover frequency (TOF) reaching 4.97 mmol g^−1^ min^−1^, demonstrating its high efficiency.

Moreover, N-CuBTC exhibited outstanding reusability, maintaining its catalytic performance across several reaction cycles, underscoring its practical potential for sustainable applications. Looking ahead, future studies could focus on further refining the synthesis techniques and exploring the applicability of the hierarchical porous catalyst in various chemical transformation processes. Specifically, transformations such as the selective oxidation of alcohols to aldehydes and ketones could benefit from the increased accessibility provided by the hierarchical pore structures. Additionally, the catalyst may be advantageous in the catalytic conversion of biomass-derived compounds, which often require efficient mass transfer due to the larger size of substrates. Furthermore, applications in coupling reactions, such as Suzuki or Heck reactions, could also leverage the unique properties of hierarchical pores to enhance reaction rates. These potential applications highlight the versatility of the catalyst and its promising role in advancing catalytic efficiency across different reaction types.

## Figures and Tables

**Table 1:** Pore structure information for M-CuBTC and N-CuBTC.

Sample	Yield /(%) ^a^	Particle Size /(nm) ^b^	*S*_BET_ /(m^2^ g^−1^) ^c^	*S*_Langmuir_ /(m^2^ g^−1^)	*V*_pore_ /(cm^3^ g^−1^) ^d^
M-CuBTC	90.2	~12000	1290	3150	0.47
N-CuBTC	93.1	~50	1220	2990	0.58

^a^ The yield is calculated based on a copper to BTC ratio of 1.67:1. ^b^ The average particle size was assessed using Gaussian statistical methods, with the number in parentheses indicating the standard deviation. ^c^ The surface area was determined from N_2_ adsorption isotherms at 77 K within the pressure range of 0.05 < P/P_0_ < 0.2, with an estimated standard deviation of ±50 m^2^ g^−1^. ^d^ The pore volume was derived from N_2_ adsorption isotherms at 77 K, specifically at P/P_0_ = 0.95, for pores measuring ≤ 20 nm.

## Data Availability

The original contributions presented in this study are included in the article. Further inquiries can be directed to the corresponding author.
